# Effective Carbon/TiO_2_ Gel for Enhanced Adsorption and Demonstrable Visible Light Driven Photocatalytic Performance

**DOI:** 10.3390/gels8040215

**Published:** 2022-04-01

**Authors:** Anam Safri, Ashleigh Jane Fletcher

**Affiliations:** Department of Chemical and Process Engineering, University of Strathclyde, Glasgow G1 1XJ, UK; anam.safri@strath.ac.uk

**Keywords:** adsorption, carbon/TiO_2_ gels, resorcinol formaldehyde RF/TiO_2_ gels, photocatalysis, adsorption kinetics, methylene blue dye degradation

## Abstract

A new strategy to synthesise carbon/TiO_2_ gel by a sol–gel method is proposed. Textural, morphological, and chemical properties were characterised in detail and the synthesised material was proven to be an active adsorbent, as well as a visible light photocatalyst. Homogenously distributed TiO_2_ is mesoporous with high surface area and, hence, exhibited a high adsorption capacity. The adsorption equilibrium experimental data were well explained by the Sips isotherm model. Kinetic experiments demonstrated that experimental data fitted a pseudo second order model. The modification in electronic structure of TiO_2_ resulted in a reduced bandgap compared to commercial P25. The absorption edge studied through UV-Vis shifted to the visible region, hence, daylight photocatalytic activity was efficient against degradation of MB dye, as an example pollutant molecule. The material was easily removed post treatment, demonstrating potential for employment in industrial water treatment processes.

## 1. Introduction

Adsorption of carbon is perhaps the most widely used water treatment technique. However, there is an ongoing effort to develop efficient adsorbents with reduced regeneration costs. Currently, the combination of carbon and titanium dioxide (TiO_2_) appears to offer a promising route to obtain an adsorbent with self-regeneration properties. Additionally, the synergistic effect of both carbon and TiO_2_ enhances the degradation process due to respective adsorptive and photocatalytic properties. Literature reports several studies to address the synergy of adsorption and photodegradation by experimental demonstration of various carbon/TiO_2_ composite materials [1,2]. However, there is still a need to better understand the phenomenon of pollutant-adsorbent interactions in order to have a good knowledge to design an efficient water treatment process. Additionally, the improvement in design involves the type of materials and synthesis process employed to attain maximum efficiency of the system.

Previously, carbon has been combined with TiO_2_ through various approaches, in different forms, such as carbon nanotubes [3,4,5], graphene [6,7,8], and activated carbon [9,10]. Lately, focus has been shifted to highly porous carbon materials as support matrix for industrial applications, due to the high surface area and tuneable porosity. Ideally, well-developed mesoporous structures with large pore volumes and uniform pore size distributions are preferred, due to enhanced accessible surface sites contributing to superior adsorption capacity of pollutants from the aqueous phase. However, the preparation process of these mesoporous carbons is costly and complicated, usually resulting in materials with moderate or low surface area. The efficiency of the material is also limited, since most TiO_2_ nanoparticles incorporated in the pores of the carbon are unavailable for photocatalysis [11].

Amongst mesoporous carbon materials, carbon gels are a new type of nanocarbon with potential applications in photocatalysis [2,12,13]. Carbon gels produced by polycondensation of resorcinol (R) with formaldehyde (F) are highly porous and have flexible properties. A comprehensive review of sol–gel synthesis of RF gel reveals that the material can be easily tailored to attain desired properties, mainly tuneable porosity, and acts as a support for metals [14]. Hence, RF gels can be promising materials for water treatment applications, mainly due to their stability, owing to aromatic resorcinol rings and their overall interconnected mesoporous carbon structure. For industrial applications where a continuous process system is often required, carbon derived from RF gels can be more efficient and cost-effective than commercial adsorbents, which are in the form of granules or powders and are unsuitable for use in continuous systems. 

The aim of this study is to synthesize an adsorbent with visible light driven photocatalytic activity by incorporating TiO_2_ nanoparticles into RF gels. A typical synthesis route of an RF gel [15] is modulated in this study to integrate TiO_2_ nanoparticles by formulating a twostep synthesis scheme. In addition to enhancement in textural properties of this newly synthesised adsorbent, improvement in photocatalytic properties is expected by (i) modification in electronic structure of TiO_2_, due to the presence of RF gel as a carbon source, shifting the absorption edge to the visible light region, hence, enabling TiO_2_ to activate under visible light irradiation; (ii) the carbon phase can entrap the photogenerated electron and hole pairs, which would otherwise recombine and dissipate heat energy; and (iii) the porous RF gel helps facilitate dispersion of TiO_2_ and easy post treatment removal of the adsorbent/photocatalyst. 

Here, we report a study of the textural and optical characteristics of the adsorbent/photocatalyst. Detailed adsorption experiments were carried out to study the effect of several parameters on adsorption capacity. Additionally, the interaction behaviour between potential pollutants and the material were investigated, using methylene blue (MB) as a model adsorptive. Equilibrium sorption data were modelled using Langmuir, Freundlich, Sips, and Toth isotherm models. Kinetic analyses were carried out by comparing the experimental data with pseudo first order and pseudo second order expressions, as well as a diffusion model to better understand the transfer behaviour of the adsorbate species. Further, photocatalytic application tests were performed under visible light irradiation and the data were modelled to study the kinetics of photocatalysis. 

## 2. Results and Discussion

### 2.1. Morphology

**Figure 1:**
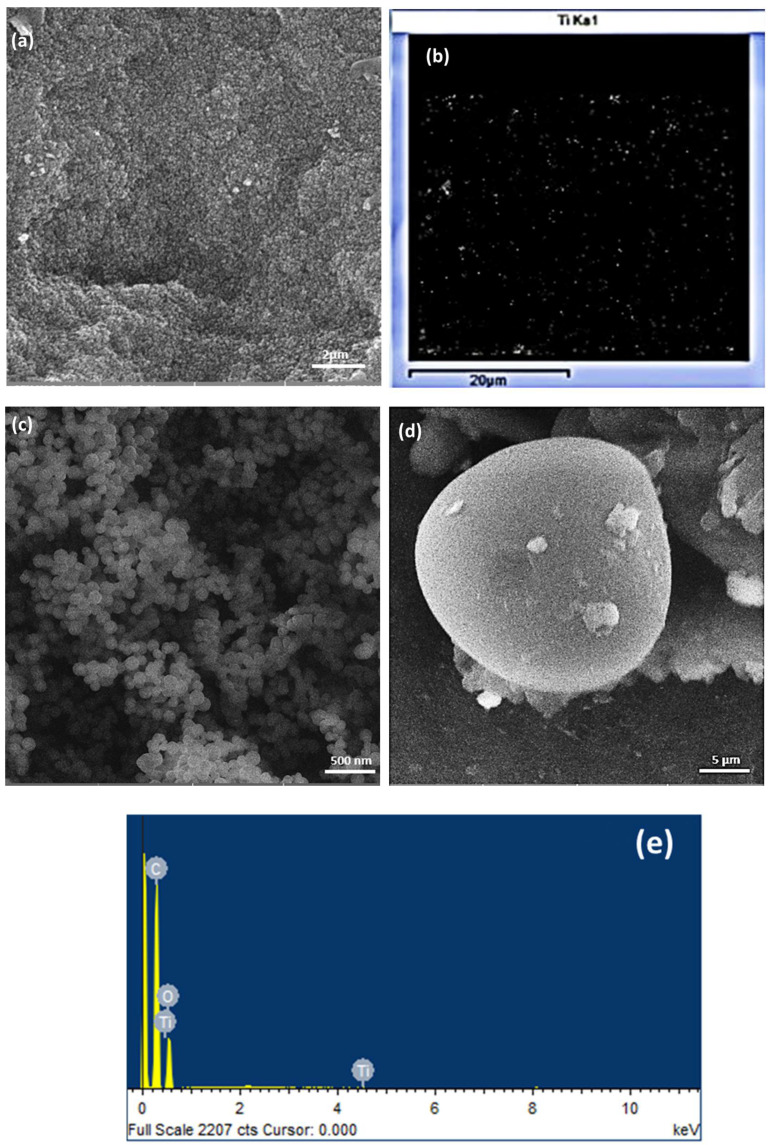
Morphology of RF/TiO_2_ sample (**a**) FESEM image of RF/TiO_2_, (**b**) TiO_2_ distribution determined by EDX on the sample, (**c**) distinct appearance of micro/nanospheres, (**d**) isolated microsphere with differentiation between organic–inorganic phase, and (**e**) corresponding EDX spectra.

The morphology of sample, studied using FESEM, is shown in Figure 1. Figure 1a shows a heterogenous nature of synthesised RF/TiO_2_ with homogenously distributed TiO_2_, as represented in Figure 1b. The overall structure shows the nanospheres connected to form a three-dimensional porous network, as represented in Figure 1c [14]. The heterogenous surface is more evident in Figure 1d where organic and inorganic phases can be differentiated. The diameter of microspheres ranged around 0.76–1.66 μm, indicating that the size of the primary particles was slightly larger than pristine RF, which generally is in the nanometre range [16]. Energy dispersive X-ray (EDX) spectra of the microspheres is shown in Figure 1e, (EDX zone shown in supplementary information, Figure S1) which evidently corresponds to the recorded spectra.

### 2.2. FTIR Analysis

**Figure 2:**
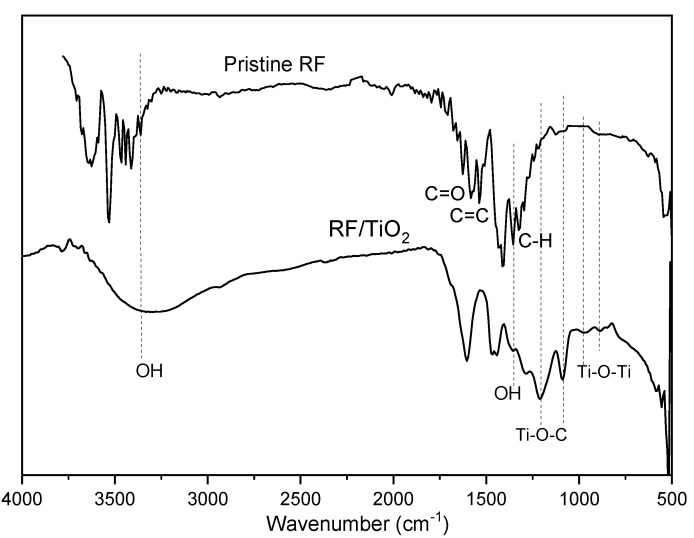
FTIR spectra of synthesised RF/TiO_2_ gel compared to pristine RF gel.

The IR absorption bands of RF/TiO_2_ overall resembled those of the pristine RF gel, as also observed through FESEM images with clear uniform spheres illustrating a porous network and the retention of the gel structure even after addition of TiO_2_. Typical characteristic peaks, such as the previously reported C=C stretching, CH_2_, and C-O-C of aromatic rings, methylene bridges, and methylene ether bridges [17,18], were observed. The broad peak at 3300 cm^−1^ is characteristic of stretching vibrations associated with phenolic OH groups. Weak vibrations in the range of 2000–1700 cm^−1^ are attributed to CH bending of aromatic compounds. The absorption bands at 1605 and 1473 cm^−1^ correspond to aromatic ether bridges, attributed to condensation of resorcinol to form the RF gel network. A strong IR peak, expected in the range 1740–1700 cm^−1^, associated with C=O stretching of aldehyde, was not observed, which confirms that the sol–gel reaction was complete. In comparison to a spectrum of pristine RF, a few additional peaks were observed that verify the chemical linkages between RF and TiO_2_, as marked in Figure 2. It has been established that the oxygenated surface groups of carbon materials support the attachment of TiO_2_. [19]. Here, crosslinking of TiO_2_ with RF, via the hydroxyl groups, can be observed through the peaks in the vicinity of 1400 cm^−1^, attributed to OH groups of RF, which appeared weak in the spectrum of RF/TiO_2_, signifying the reaction of OH and TiO_2_. Meanwhile, new signals observed at 1200 and 1084 cm^−1^ suggest formation of Ti-O-C functionalities. Similar crosslinking has previously been reported in TiO_2_/phenol resol hybrid structures, where chemical interactions between TiO_2_ and phenol resol form Ti-O-C complexes. This heterojunction is responsive to visible light due to formation of a charge complex between the interface of TiO_2_ and mesoporous phenol resol producing new electronic interactions [20]. Hence, it can be concluded that the interactions between RF and TiO_2_ are chemical in nature. Additional signals below 1000 cm^−1^, such as bands at 963 and 880 cm^−1^, are associated with titanium ethoxide functional groups. Additionally, the broad band observed in the range of 600 cm^−1^ corresponds to the vibration of Ti-O-Ti bonds [21]. 

### 2.3. Surface Area Analysis

**Figure 3:**
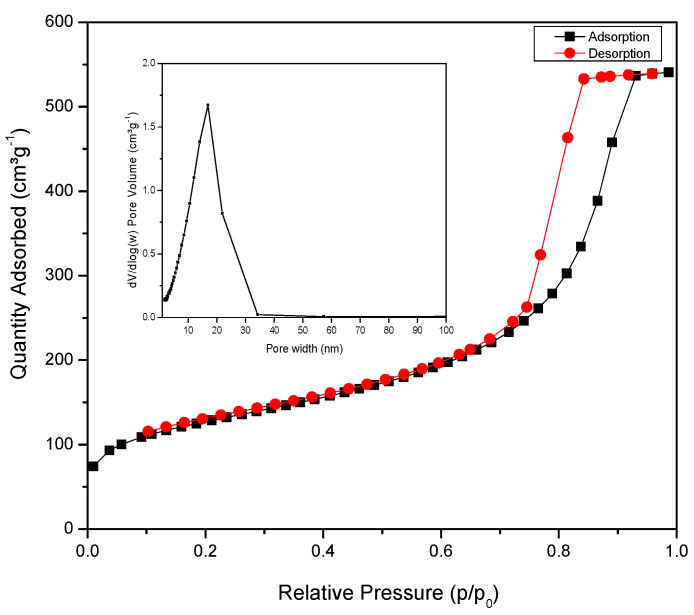
Nitrogen sorption isotherms and pore size distribution of synthesised RF/TiO_2_ gel.

A nitrogen sorption isotherm was measured to determine the specific surface area and pore volume of RF/TiO_2_. Figure 3 shows N_2_ sorption isotherm and pore size distribution (inset Figure 3). As can be seen, the isotherm of RF/TiO_2_ is of Type IV classification [22] with a sharp capillary condensation at P/P_o_ = 0.4–0.9 and a well-defined hysteresis loop of Type H1, associated with open ended pores whilst suggesting a mesoporous structure [16]. Pore filling occurs at low relative pressure and the calculated mesoporosity in the structure was ~94%. The S_BET_, corresponding pore size and total pore volume of as prepared RF/TiO_2_ is 439 m^2^ g^−1^, 9.4 nm and 0.71 cm^3^ g^−1^, respectively. The S_BET_ value of pristine RF gel obtained in this study is 588 m^2^ g^−1^. The reason in reduced S_BET_ value for RF/TiO_2_ is attributed to blockage of pores of RF gel matrix with inclusion of TiO_2_ nanoparticles. Meanwhile, in comparison with pristine TiO_2_, the S_BET_ value is significantly higher for the synthesised RF/TiO_2_. Additionally, noteworthy S_BET_ value for pristine TiO_2_ (i.e., 111 m^2^ g^−1^) is obtained in this study, contrary to commercial P25 with S_BET_ value of 57 m^2^ g^−1^.

### 2.4. Effect of pH

**Figure 4:**
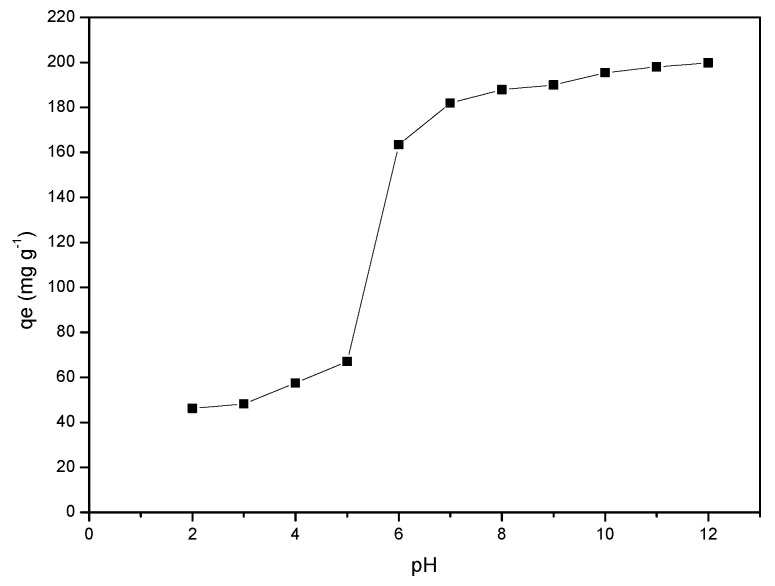
Effect of pH on the adsorption of MB dye by RF/TiO_2_ gel.

The influence of MB sorption was studied by varying the solution pH from 2–12 (25 mL, 100 mg L^−1^, 0.01 g of adsorbent). The adsorption capacities at different pH values are shown in Figure 4. The efficiency of uptake increases from 47.24 to 65.96 mg g^−1^ when the pH increases from 2–5. Thereafter, a sharp increase in adsorption capacity is observed at pH ≥ 6. The variation in adsorption behaviour of MB on RF/TiO_2_ can be explained by considering the structure of MB and evaluated point of zero charge (pzc). The pHpzc value for RF/TiO_2_ is determined to be 7.2 (Figure S2).

RF/TiO_2_ can be amphoteric having both positively and negatively charged surface sites in aqueous solution due to the varying amount and nature of surface oxygen [23]. At pH lower than the pHpzc, the surface of RF/TiO_2_ is positively charged, which repels the cationic dye (MB), and resultant interactions are hindered in acidic media due to electrostatic repulsion between the competing H^+^ ions on the surface of adsorbent and MB dye molecules. As the pH increases, the surface of RF/TiO_2_ becomes deprotonated and the adsorption sites available for interaction with cationic species increase, therefore, increased adsorption capacity is observed. This suggests that the electrostatic forces of attraction between MB and the surface of RF/TiO_2_ increases due to increased ion density and positive charges on the surface. Further, the OH groups on the surface of RF/TiO_2_ can also attract MB dye molecules under higher pH conditions. 

Overall, a good adsorption capacity for MB is observed at pH higher than the pHpzc due to an increased number of negative sites in the higher pH range. This is in good agreement with the fact that, due to the presence of COO^−^ and OH- functional groups, MB dye adsorption is favoured at pH > pHpzc [24]. The same trend has been observed in previous studies with activated carbon and TiO_2_ composites where reduced activity was observed at acidic pH and maximum activity was observed in the pH range 6–10 [25,26,27].

### 2.5. Effect of Contact Time

**Figure 5:**
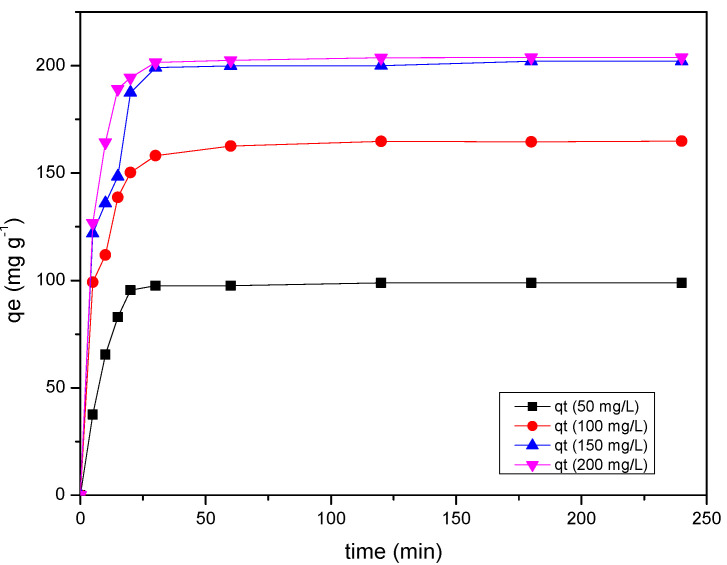
Effect of adsorption on contact time and initial concentration of MB dye by RF/TiO_2_ gel.

Figure 5 shows the effect of contact time on the amount of MB molecules adsorbed by RF/TiO_2_ gel under different initial MB concentrations. As shown, the adsorption capacity increases with increase in initial concentration. The equilibrium adsorption capacity increases from 102 mg g^−1^ to 207 mg g^−1^ by increasing the initial concentration of MB from 50 mg L^−1^ to 200 mg L^−1^. Initially, the adsorption capacity overall is rapid for timeframes up to 30 min. This trend is expected, due to the greater driving force of MB dye molecules and immediate availability of vacant adsorption sites, hence resulting in increased in frequency of collisions between MB dye molecules and the RF/TiO_2_ gel. Additionally, mesoporosity throughout the RF/TiO_2_ gel structure provides a high surface area for greater adsorption of MB molecules. It is noteworthy that at higher MB concentration the adsorption rate is greater and adsorption capacity attains equilibrium faster than at low concentration. The reason is attributed to immediate occupancy of available active sites by a large amount of adsorbate molecules. This rapid occurrence of sorption is due to the presence of mesoporosity within the RF/TiO_2_ gel, which corresponds to a large portion of the adsorption sites. In this case, the mesoporous structure provides a large surface area to solution volume within the porous network of the adsorbent gel. Additionally, within the mesopores, MB dye molecules are confined to be in close proximity to the surface. Such observations have been reported in previous research, particularly for activated carbons [28]. Over time, saturation of active sites occurs, and adsorption becomes difficult on the fewer available active sites due to repulsive forces between the MB molecules and the RF/TiO_2_ gel surface. Additionally, the blockage of pores and charge repulsion of MB dye species may decelerate the adsorption progress. Similar phenomena have been explained for porous TiO_2_ and other carbon/TiO_2_ porous composite materials, where it may have taken longer for the adsorbate to diffuse deeper in the fine pores [29]. Thereafter, the adsorption capacity increases gradually until 90 min, and equilibrium is attained for the entire concentration range. Thus, equilibrium time was considered as 90 min which was considered sufficient for removal of MB ions by RF/TiO_2_ gel. Hence, the contact time was set to 90 min in the remaining experiments to ensure equilibrium was achieved.

### 2.6. Effect of Sorbent Dose

**Figure 6:**
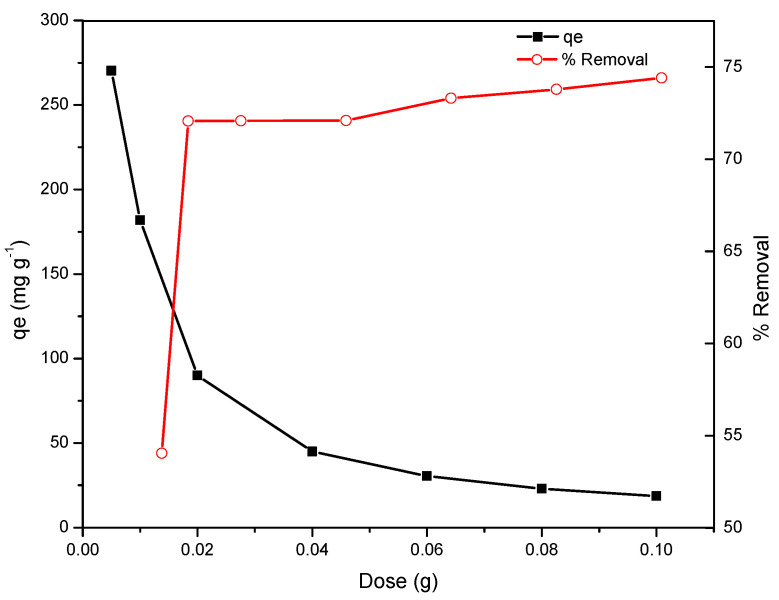
Effect of adsorbent dose on the removal and adsorption of MB dye by RF/TiO_2_ gel.

The percentage removal of MB dye increased with increase in the adsorbent dose from 0.005 to 0.01 g but remained almost constant with further increase in the dose range 0.01 to 0.1 g, as represented in Figure 6. Percentage removal was calculated using Equation (2), and showed an increase with increase in adsorbent dose, due to greater availability of vacant active sites, a large surface area, and a greater number of adsorptive sites present on the surface of RF/TiO_2_. With further increase in adsorbent dose (>0.01 g), the rate of MB removal becomes low, as the concentrations at the surface and solution reach equilibrium. The resultant reduction in adsorption rate is attributed to unoccupied adsorbent sites, as well as overcrowding or aggregation of adsorbent particles [30]. Hence, the surface area available for MB adsorption per unit mass of the adsorbent reduces, whereby percentage removal was not significantly enhanced with further increase adsorbent dose.

### 2.7. Adsorption Kinetics

**Figure 7:**
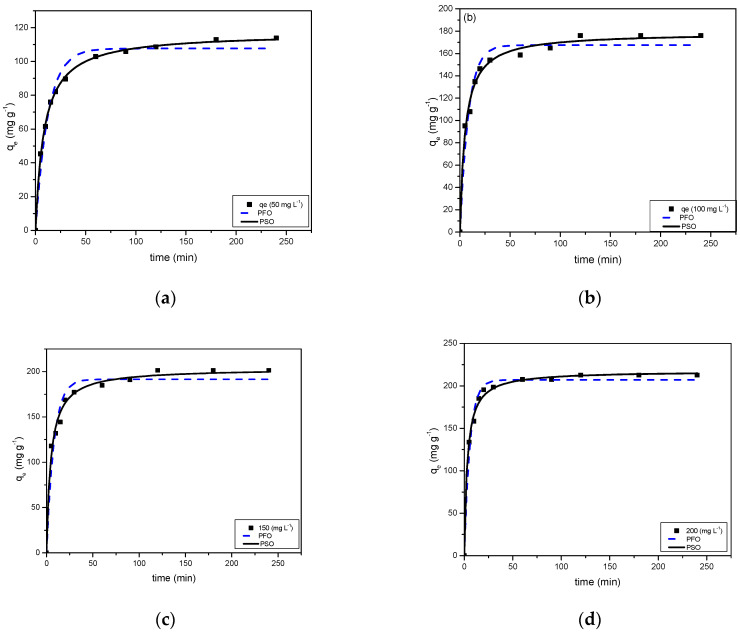
MB uptakes on RF/TiO_2_ gel at (**a**) 50 mg L^−1^, (**b**) 100 mg L^−1^, (**c**) 150 mg L^−1^, (**d**) 200 mg L^−1^, and fitted data for pseudo first order and pseudo second order kinetic models.

The adsorption kinetics were studied using a contact time of 240 min in the concentration range 50–200 mg L^−1^. The experimental data obtained for MB dye adsorption capacity vs. time (t) were fitted with PFO and PSO, as presented in Figure 7a–d. The parameters determined, including measured equilibrium adsorption capacity qe (experimental), theoretical equilibrium adsorption capacity qe (calculated), first order rate constant K_1_, second order rate constant K_2_, and regression coefficient R^2^, are presented in Table 1.

As observed from the data, the correlation factor R^2^ deviates significantly from 1 for PFO and, therefore, pseudo first order model does not exhibit good compliance with the experimental data for the entire concentration range. This implies that the adsorption reaction is not inclined towards physisorption, and the MB dye molecules adsorb to specific sites on the surface of RF/TiO_2_ gel. The argument regarding the failure of the pseudo first order model suggests that several other interactions are responsible for the sorption mechanism. Hence, the correlation coefficients R^2^ of the pseudo second order model were compared with pseudo first order parameters. R^2^ values for pseudo second order behaviour are approximately 0.99 for the entire concentration range, indicating that the system is more appropriately described by the pseudo second order equation. The dependence on initial concentration of MB dye is verified by good compliance of the experimental data with the pseudo second order equation, where the adsorption capacity is affected by the initial MB dye concentration, subsequent surface-active sites, and adsorption rate (Other error analyses are represented in Table S1).

The equilibrium sorption capacity increased from 116.97 to 217.59 mg g^−1^ when initial dye concentration was increased from 50 to 200 mg g^−1^ confirming that MB dye removal is dependent on initial concentration, where the rate limiting step is determined by both adsorbate (MB) and adsorbent (RF/TiO_2_) concentration. This signifies that the sorption mechanism is chemisorption. Previous studies have explained theoretically that if diffusion is not the rate limiting factor, then higher adsorbate concentrations would give a good pseudo first order fit whereas, for low concentrations, pseudo second order better represents the kinetics of sorption, analogous to the observations made here [31]. Previously, the adsorption processes of MB on TiO_2_/carbon composites have also exhibited strong dependencies of pseudo second order fitting parameters on initial concentrations [27]. 

**Figure 8:**
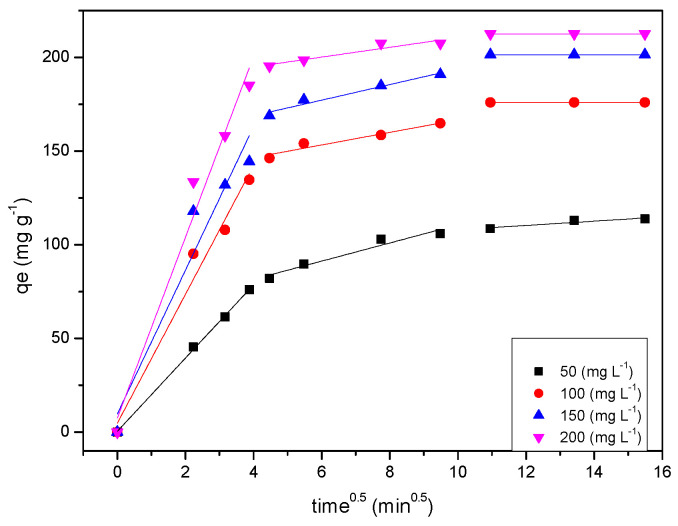
Intra-particle diffusion kinetics of MB dye adsorption on RF/TiO_2_.

Figure 8 shows a plot of MB dye uptake (qe) on synthesised RF/TiO_2_ against (time)^0.5^. The plots exhibit multi-linearity, rather than two straight lines, indicating that the adsorption process is influenced by several steps. The initial segment of the plots shows that diffusion across the boundary of the adsorbent only lasts for a short time in comparison to the whole adsorption process. This second section is attributed to diffusion into the mesopores of the adsorbent, i.e., the MB dye molecules enter less accessible pore sites. Resultantly, the diffusion resistance increases, and the diffusion rate decreases. This stage is a slow and gradual stage of the adsorption process. The third segment represents the final equilibrium stage where intra-particle diffusion slows down to an extremely low rate due to the remaining concentration of the MB dye molecules in the solution. This implies a slow transport rate of MB dye molecules from the solution (through the gel–dye solution interface) to available sites. Here, the surface of the RF/TiO_2_ gel, and micropores, may be responsible for the uptake of MB dye molecules.

### 2.8. Adsorption Isotherms

**Figure 9:**
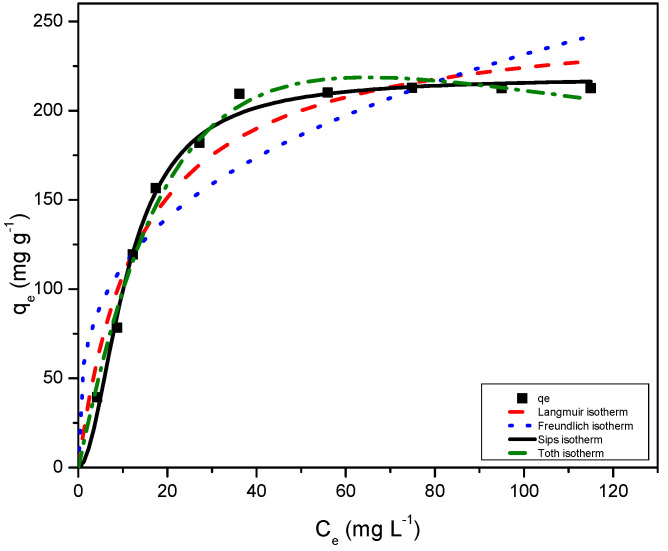
Adsorption data for RF/TiO_2_ onto MB dye corresponding fits to Langmuir, Freundlich, Sips, and Toth equation.

The equilibrium data were analysed using Langmuir, Freundlich, Sips, and Toth isotherm equations to obtain the best fit. The isotherm data plots, and fitting model parameters are shown in Figure 9 and Table 2, respectively. Comparison of the correlation factor R^2^ indicates that qe,exp fitted well to the Sips model with the lowest χ^2^ value. The qe,cal value, calculated using the Sips model, is closest to qe,exp with R^2^ closest to 1. The Sips model is a combination of the Langmuir and Freundlich adsorption isotherms, hence, the model suggests both monolayer and multilayer adsorption. At low MB dye concentrations, the model predicts Freundlich adsorption isotherms as a heterogenous adsorption system and localised adsorption without adsorbate–adsorbate interactions, whereas at high concentrations the model predicts monolayer adsorption as in Langmuir isotherm [32,33]. In the present study, the value of constant ns from Equation (11), the heterogeneity factor, is greater than 1 (i.e., n_s_ = 1.91), hence, the adsorption system is predicted to be heterogenous [33]. Further, the Toth isotherm model validates multilayer and heterogeneous adsorption, where the factor n_T_ determines heterogeneity. Here, again the value of n_T_ is greater than 1, and, therefore, the system confirms heterogeneity. It is evident that the equilibrium uptakes follow the Sips model according to the correlation factor R^2^ (other error analyses are represented in Table S2) and the isotherm models fit the data in the order Sips > Toth > Langmuir > Freundlich. 

### 2.9. Thermodynamic Study

Thermodynamic parameters for the adsorption system are recorded in Table 3. Negative values of free energy changes are evident from the data, which signifies the spontaneous adsorption of MB dye molecules on the sample for the studied temperature range. Adsorption capacity increases with an increase in temperature and a positive ∆H⁰ (Table 3) suggests that the adsorption is endothermic in nature. Positive ∆S⁰ indicates some structural changes in the MB dye and RF/TiO_2_ gel causing an increase in the degree of freedom of the MB dye species and consequently increased randomness at the adsorbent–adsorbate interface. At high temperature, the release of high-energy desolvated water molecules from the MB dye molecules and/or aggregates arise after adsorption on RF/TiO_2_ gel, which relates to a positive ∆S⁰ [34]. Before sorption begins, the MB ions are surrounded by highly ordered water clusters strongly bound via hydrogen bonding. Once MB ions come in close contact with the surface of RF/TiO_2_, the interaction results in agitation of the ordered water molecules, subsequently increasing the randomness of the system. Although, the adsorption of MB dye onto RF/TiO_2_ gel may reduce the freedom of the system, the entropy increase in water molecules is much higher than the entropy decrease in MB ions. Therefore, the driving force for the adsorption of MB on RF/TiO_2_ is controlled by an entropic effect rather than an enthalpic change. Similar phenomena have previously been reported in order to explain the fact that thermodynamic parameters are not only related to the properties of the adsorbate but also to the properties of other solid particles [35,36]. 

## 3. Photocatalytic Tests

**Figure 10:**
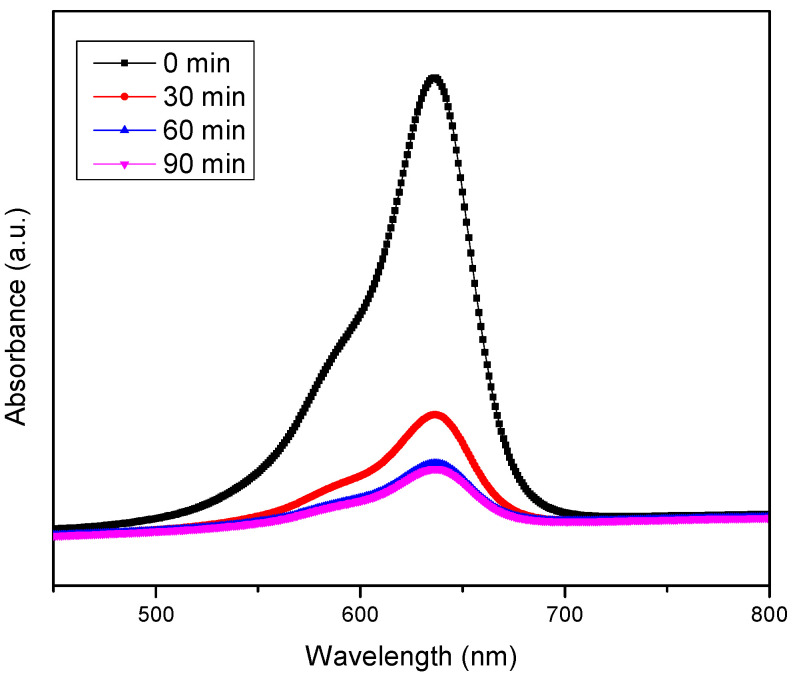
UV-Vis spectra of MB dye degradation using RF/TiO_2_ gel.

Photocatalytic activity was determined by testing the efficiency of RF/TiO_2_ against degradation of methylene blue (MB) under visible light irradiation. The maximum absorbance vs. wavelength spectra (in the range of 550–700 nm) were collected and subsequent activity, after 30 min, intervals was recorded, as shown in Figure 10. 

Within the studied systems, no photodegradation activity (reduction in concentration and decolourisation of MB dye) was observed in the absence of adsorbent/catalyst, as well as in the presence of pristine RF, indicating that the properties of MB are more stable. Additionally, RF solely may not be recommended for photocatalysis due to slow charge transfer properties, which has also been proven by the study carried out by Zang, Ni, and Liu, where the researchers employed pristine RF resins for visible light photocatalysis [37]. Slight photodegradation is observed in the presence of pristine TiO_2_, which may be attributed to the potential absorbance of UV-Vis light from the surroundings confirming that the process of MB degradation is light driven. Although the TiO_2_ obtained for use in this study has a high surface area, which may possess good adsorption properties to exhibit efficient adsorption of MB dye, since TiO_2_ only activates upon UV light irradiation (~280 nm), it does not produce enough reactive oxide species (ROS) to be an effective photodegradation system [38].

**Figure 11:**
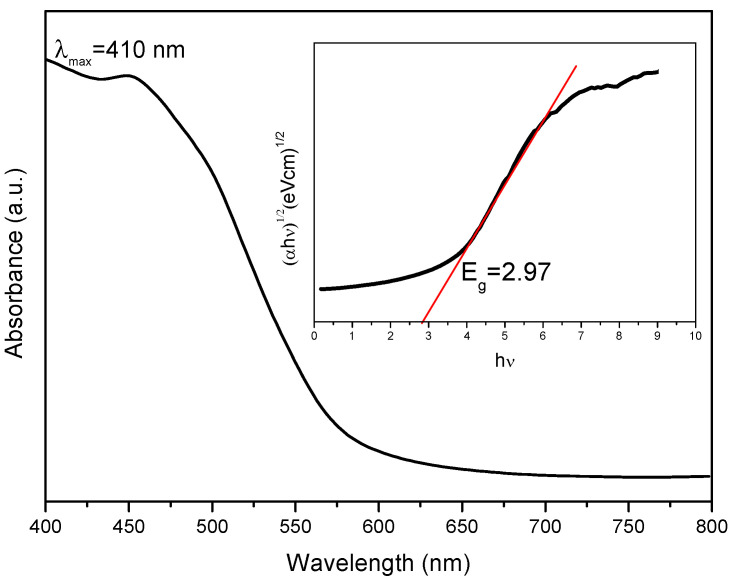
Absorption vs. wavelength spectrum of RF/TiO_2_ dispersed in ethanol measured through UV-Vis spectrophotometer, inset shows calculated band gap of synthesised RF/TiO_2_.

The dye degradation data obtained after treatment with RF/TiO_2_ showed ~73% MB dye removal after 90 min. This is attributed to the synergy of RF and TiO_2_, enabling an absorption shift to a higher wavelength, as λmax is detected at 410 nm (Figure 11). Further analysis indicates modification in the electronic structure and a subsequent reduction in bandgap occurs due to doping of TiO_2_ similar to when combined with carbon [39]. The calculated band gap energy is 2.97 eV, as shown in Figure 11 (inset). The value achieved is significantly lower than pristine TiO_2_ (i.e., 3.2 eV [21]), indicating photodegradation of MB dye under visible light irradiation. The RF matrix enables entrapment of a photogenerated electron and hole pairs and, therefore, rapid generation of ROS is possible for efficient degradation of the MB dye. These findings are comparable to other carbon/TiO_2_ systems where synergistic effects have substantially enhanced the performance of the system due to improved optical properties of the material [1,40,41].

**Figure 12:**
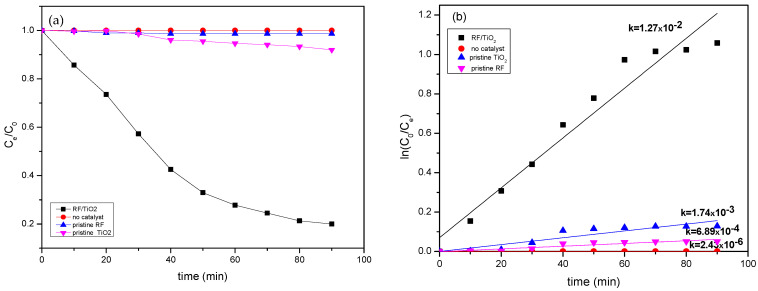
(**a**) Photocatalytic performance regarding MB dye degradation without catalyst, and with pristine RF, pristine TiO_2_ and RF/TiO_2_ gel (**b**) First-order kinetics of photoactivity without catalyst, and with pristine RF, pristine TiO_2_, and RF/TiO_2_ gel.

The photodegradation of MB dye, that is, the reduction in concentration with time is recorded in Figure 12 and the recorded data is modelled using pseudo first order kinetics, shown in Figure 12a,b. The data are fitted to the first order kinetic equation (ln (C_o_/Ce) = kt) to evaluate the value of the rate constant by slope of plot ln(C_o_/Ce) vs. time (t) in minutes, where C_o_ and Ce is the initial at t = 0 and final concentration at given time of MB concentration, respectively. The value of k here is the measure of photocatalytic performance, as it defines the concentration of reacting substances, that is, photogenerated reactive oxide species, therefore, a higher value of k signifies higher photocatalytic efficiency. As compared to no catalyst (k = 2.43 × 10^−6^ min^−1^) pristine TiO_2_ (k = 1.74 × 10^−3^ min^−1^) and pristine RF (k = 6.89 × 10^−4^ min^−1^), the rate of RF/TiO_2_ was the highest (k = 1.27 × 10^−2^ min^−1^). Clearly, the rate constant obtained for photodegradation of MB using RF/TiO_2_ was the highest. Mainly, improved optical property was the most important advancement in forming RF/TiO_2_ gel which is photocatalytically active under visible light (410 nm) irradiation. 

The RF/TiO_2_ material created in this study exhibits excellent photoactivity under visible light, which can further be explained by the mechanism of MB photodegradation represented in Equations (1)–(8). The system activates when RF/TiO_2_ absorbs light with photon energy (hν) and generates conduction band (CB) electron (e^−^) and valence band (VB) hole (h^+^) pairs upon under visible light irradiation. The holes interact with moisture on the surface of the adsorbent gel yielding hydroxy free radicals or reactive oxide species (H^+^ or OH^●^), which are oxidation agents that can mineralise a wide range of organic pollutants, ultimately producing CO_2_ and H_2_O as end products. The reaction sequence below represents the photodegradation of MB, showing a simplified mechanism of photoactivation by a photocatalyst (Equations (1)–(4)) [19]. For the mechanism of photoinactivation of MB in the presence of RF/TiO_2_, hydroxy free radicals or reactive oxide species (H^+^ or OH^●^) attack the aromatic ring of the MB structure, degrading it into a single ring structure product, which then finally degrades to CO_2_ and H_2_O (Equations (5)–(8)) [42,43].

(1) $$\mathrm{RF}/{\mathrm{TiO}}_{2}+h\nu =e_{\mathrm{CB}}^{-}+h_{\mathrm{vB}}^{+}$$

(2) $$h_{\mathrm{CB}}^{-}+{\;H}_{2}O=H^{+}+{\mathrm{OH}}^{˙}$$

(3) $$e_{\mathrm{CB}}^{-}+O_{2}=O_{2}^{.-}$$

(4) $$O_{2}^{.-}+H^{+}={\mathrm{HO}}_{2}^{.}$$

(5) $$\mathrm{MB}+\;\mathrm{RF}/{\mathrm{TiO}}_{2}={\mathrm{MB}}^{.+}+{\;e}_{\mathrm{CB}}^{-}\left(\mathrm{RF}/{\mathrm{TiO}}_{2}\right)$$

(6) $$O_{2}+e^{-}=O_{2}^{.-}$$

(7) $${\mathrm{MB}}^{.+}+{\mathrm{OH}}^{-}=\mathrm{MB}+{\mathrm{OH}}^{˙}$$

(8) $${\mathrm{MB}}^{.+}+{\mathrm{OH}}^{.}=H_{2}O+{\mathrm{CO}}_{2}+\;\mathrm{other}\;\mathrm{products}$$

## 4. Conclusions

An RF/TiO_2_ gel was successfully synthesised using sol–gel techniques via a straightforward route. The synergy of RF and TiO_2_ exhibited excellent adsorption–photodegradation activity due to the corresponding characteristics, mainly mesoporosity and photocatalysis. The synergy of contributing materials allowed modification in the electronic structure of TiO_2_ by formation of Ti-O-C chemical linkages, responsible for a reduction in the band gap of TiO_2_ for photodegradation upon visible light irradiation. Kinetic studies revealed a pseudo second order reaction, signifying chemisorption phenomenon is involved in the adsorption mechanism. The adsorption isotherm study showed that the system was heterogeneous following the Sips model equation. The spontaneity of the process was validated via thermodynamic studies, which signified an entropically driven adsorption mechanism. Effective photodegradation results were observed due to the high adsorption capacity and improved optical properties of the material, enabling significant MB dye degradation within 90 min. Overall, the material possesses properties that have potential to effectively reduce/eliminate a wide range of pollutants and, therefore, can be employed as a low-cost photocatalytic adsorbent for water treatment Especially in the industrial applications where post treatment separation and recovery of the adsorbent is difficult, employing this material can reduce the costs since in this case the adsorbent precipitates and easy separation is possible just by filtration or even decantation. 

## 5. Material and Methods

### 5.1. Synthesis 

Synthesis of RF and TiO_2_ precursors was carried out in two separate systems, which were integrated and processed further in order to obtain the final gel. 

System 1: Preparation of Titania Sol

For preparation of the titania sol, 1.78 g of titanium precursor: titanium isopropoxide (TTIP) (98+%, ACROS Organics™, Geel, Belgium) was dissolved in ethanol and stirred for 30 min. A mixture of water and HCl was added dropwise to the titania/EtOH solution under constant stirring, at room temperature, to begin hydrolysis. After 2 h, a homogenous solution was obtained. The molar ratios for these parameters were 1 TTIP:10 EtOH:0.3 HCl:0.1 H_2_O. 

System 2: Preparation of RF sol

In total, 7.74 g of resorcinol (SigmaAldrich, ReagentPlus, 99%, Poole, UK) was added to 50 mL of deionised water until completely dissolved. 0.0249 g of sodium carbonate (Na_2_CO_3_, Sigma-Aldrich, anhydrous, ≥99.5%), as a catalyst, and 4.23 g of formaldehyde (37wt%) were added to the dissolved resorcinol under continuous stirring, at room temperature.

Finally, the prepared titania sol (system 1) was gradually transferred to the RF sol (from system 2) under constant stirring, at room temperature. The resulting sol was stirred at room temperature for 2 h after which the sol mixture was aged at 85 °C for 72 h.

After aging, the process of solvent exchange and drying the RF/TiO_2_ gel, first involved cutting the gel into smaller pieces. These pieces were then immersed in acetone for 72 h to facilitate solvent exchange prior to drying, followed by vacuum drying at 110 °C for 48 h to obtain the final RF/TiO_2_ adsorbent gel. In this way, the final gel corresponded to 10 wt% TiO_2_ (theoretical percentage) incorporated in the RF gel matrix.

### 5.2. Adsorbent Characterisation

Morphology of the synthesised sample was studied by field emission electron scanning microscope (FESEM) TESCAN-MIRA. The functional groups on the surface of synthesised RF/TiO_2_, and the chemical linkages between the constituent RF and TiO_2_ components, were verified using Fourier Transform Infrared Spectroscopy (FTIR) (MB3000 series, scan range 4000–400 nm). BET surface area measurements were carried out using a Micromeritics ASAP 2420 to obtain N_2_ adsorption isotherm at 77 K and pore size was determined via BJH theory [22]. A UV-Vis Spectrophotometer (Varian Cary 5000 UV-Vis NIR Spectrophotometer Hellma Analytics) was used to collect absorption spectra and the data used to interpret the change in electronic structure of RF/TiO_2_ [44]. The data were manipulated to calculate the band gap energy values through the Tauc method described in previous studies [44].

## 6. Adsorption Experiments

### 6.1. Effect of pH

The effect of pH on the sorption of methylene blue (MB) dye was investigated with 0.01 g of sample by adjusting the pH of solution (25 mL, 100 mg L^−^^1^ MB) between 2 and 12, at 23 °C. The pH was adjusted using 0.01 M HCl and 0.01 M NaOH. After 2 h of agitation, the solution was centrifuged for 15 min and the supernatant solution was collected via syringe. The initial and final concentrations were measured using a UV-Vis spectrophotometer (Varian Cary 5000 UV-Vis NIR Spectrophotometer, Agilent, UK) and onward calculations were performed. 

### 6.2. Effect of Sorbent Dose

The amount of sorbent dose was gradually increased from 0.005 to 0.01 g to study the effect of sorbent dose on the adsorption capacity. pH and temperature were maintained at 7.0 and 23 °C, respectively, against 25 mL of 100 mg L^−^^1^ MB concentrated solution. The pH was adjusted using 0.01 M HCl and 0.01 M NaOH. After 2 h of agitation, the solution was centrifuged for 15 min and the supernatant solution was collected via syringe. The initial and final concentrations were measured using a UV-Vis spectrophotometer (Varian Cary 5000 UV-Vis NIR Spectrophotometer Hellma Analytics) and onward calculations were performed.

### 6.3. Effect of Initial Concentration

All adsorption experiments were performed at 23 °C in 125 mL conical flasks, using a shaker (VWR 3500 Analog orbital shaker) set to 125 rpm. The first experiment was conducted to study the isothermal equilibrium and the effect of initial MB concentration. Standard solutions of MB were prepared using distilled water, with initial concentrations in the range of 20–200 mg L ^−1^. Then, 25 mL aliquots were distributed into each flask, and 0.01 g of the adsorbent gel was added individually to each flask. The pH values of all solutions were recorded and adjusted to 7.0, if required, using 1 M HCl and 1 M NaOH. After 2 h of agitation, the solution was centrifuged for 15 min and the supernatant solution was collected via syringe. The initial and final concentrations were measured using UV-Vis spectrophotometer (Varian Cary 5000 UV-Vis NIR Spectrophotometer Hellma Analytics). 

The equilibrium adsorption capacity, q_e_ (mg g^−1^), was calculated using:

(9) $$q_{e}=\frac{\left(C_{o}-C_{e}\right)\times V\left(l\right)}{W}$$

while the respective percentage removal of MB was calculated as:

(10) $$\mathrm{Removal}\;\%=\frac{C_{o}-C_{e}}{C_{o}}\times 100\%$$

where C_o_ and C_e_ are the initial MB and final concentration, respectively. W is the weight (g) of the adsorbent and V is the volume (L) of MB solution.

### 6.4. Effect of Contact Time

The effect of contact time was studied by adding MB solution (pH 7.0, 25 mL, 100 mg L^−1^) and 0.01 g adsorbent gel into a flask, which was then agitated for a predetermined contact time between 5 min and 4 h. The samples were prepared and treated as described in Section 2.5 and the amount of adsorption was calculated using Equation (11):

(11) $$q_{t}=\frac{\left(C_{o}-C_{t}\right)\times V}{W}$$

where C_t_ is the equilibrium MB concentration at a given time, and C_o_, V, and W are as previously defined. Equilibrium concentration was determined by plotting $q_{t}$ versus time of aliquots collected at different time intervals. Adsorption-photodegradation (absorption) changes of MB dye with time were also recorded via UV-Vis spectrophotometry.

### 6.5. Effect of Temperature

The effect of temperature on the removal of MB (pH 7.0, 25 mL, 20–200 mg L^−1^) was investigated by adding a known concentration MB solution and 0.01 g adsorbent gel to a flask. A hot plate with a stirrer (120 rpm) was used to maintain a constant temperature of 8, 23, 32, and 40 °C, under stirring, for 120 min after which the absorbance versus wavelength spectra were recorded to measure the final concentration, and subsequent adsorption was calculated using Equation (9).

### 6.6. Kinetic Models

The kinetic-based models: pseudo first order (PFO) and pseudo second order (PSO) were applied to study the adsorption kinetics and to explain the mode of sorption of MB onto the synthesised RF/TiO_2_. The PFO model [33] has been frequently used to describe kinetic processes under non-equilibrium conditions. PFO is based on the assumption that the rate of adsorption is proportional to the driving force, that is, the difference between the equilibrium concentration and solid phase concentration, presented as a differential Equation (12):

(12) $$\frac{{\mathrm{dq}}_{t}}{\mathrm{dt}}=k_{1}(q_{e}-q_{t})$$

Integrating Equation (13) with the initial condition of q_t_ = 0 at t = 0, the PFO model can be rewritten, in a linear form, as:

(13) $$q_{t=}q_{e}\left(1-e^{-k_{1}t}\right)$$

Several studies have also reported the use of PSO [45] to interpret data obtained for the sorption of contaminants from water, including dyes, organic molecules, and metal ions. The PSO model assumes that the overall adsorption rate is proportional to the square of the driving force and can be expressed as Equation (14):

(14) $$\frac{{\mathrm{dq}}_{t}}{\mathrm{dt}}=k_{1}{\left(q_{e}-q_{t}\right)}^{2}$$

Integrating Equation (14) with the initial condition of q_t_ = 0 at t = 0, and q_t_ = t at t = t, the PSO model can be rewritten as:

(15) $$q_{t}=\frac{k_{2}{\mathrm{tq}}_{e}^{2}}{1+k_{2}{\mathrm{tq}}_{e}}$$

In Equations (13)–(15), qt (mg g^−1^) and q_e_ (mg g^−1^) are the adsorption capacities of MB dye molecules at time t and at equilibrium, respectively. k_1_ (mg g^−1^ min^−1^) and k_2_ (mg g^−1^ min^−1^) are the PFO and PSO rate constants, respectively.

### 6.7. Sorption Isotherm Models

The equilibrium data for the sorption of MB on RF/TiO_2_ adsorbent gel as a function of equilibrium concentration (C_e_ mg L^−1^) was analysed in terms of Langmuir, Freundlich, Sips, and Toth isotherm models [2]. The nonlinear form of Langmuir’s isotherm model is represented as:

(16) $$q_{e}=\frac{q_{m}K_{L}C_{e}}{1+C_{e}K_{L}}$$

where q_e_ (mg g^−1^) is the MB uptake at equilibrium, C_e_ (mg L^−1^) is the equilibrium concentration, q_m_ (mg g^−1^) is the amount of adsorbate at complete monolayer coverage, and K_L_ is the Langmuir constant.

The Freundlich equation can be expressed as follows:

(17) $$q_{e}=K_{F}C_{e}^{1/n}$$

where $q_{e}$ and C_e_ are as defined in the Langmuir equation, adsorption affinity is related to the adsorption constant K_F_, and n indicates the magnitude of the adsorption driving force and the distribution of energy sites on the adsorbent surface, if n < 1, then adsorption is a chemical process, whereas if n > 1, then adsorption maybe dependent on distribution of the surface sites. Generally, n values within 1–10 represents good adsorption [46].

The Sips isotherm model is a combination of the Langmuir and Freundlich isotherms and is represented as:

(18) $$q_{e}=\frac{q_{s}K_{s}C_{e}^{ns}}{1+K_{s}C_{e}^{ns}}$$

where $q_{e}$ and C_e_ are as defined for Equation (16), K_s_ is the Sips isotherm model constant (L g^−1^), and ns; is the Sips isotherm exponent.

The Toth model also describes heterogeneous systems, considering both low- and high-end concentrations. The Toth expression is as follows:

(19) $$q_{e}=\frac{q_{m}K_{T}C_{e}}{{\left[1+(K_{T}C_{e}){\;}^{t}\right]}^{1/t}}$$

where $q_{e}$ and C_e_ are as defined for Equation (16), $q_{m}$ is the maximum adsorption capacity, t is the surface heterogeneity, and $K_{T}\;$ is the surface affinity. 

### 6.8. Photodegradation Procedure

Photocatalytic performance of as prepared RF/TiO_2_ was investigated through MB dye degradation, by recording the dye degradation spectra with time using UV-Vis Spectrophotometry. 0.01 g of the adsorbent dose were used against 25 mL of 100 mg L^−1^ dye concentration at pH ~7 and a temperature of 23 °C and light intensity of 111 Wm^−2^. For comparison, the measurements were also recorded in the absence of catalyst, as well as pristine RF and TiO_2_. All suspensions were stirred in the dark for 60 min to establish sorption equilibrium before exposure to visible light. 

## Figures and Tables

**Table 1:** Kinetic parameters obtained by fitting kinetic data for MB adsorption to RF/TiO_2_.

Model	50 mg L^−1^	100 mg L^−1^	150 mg L^−1^	200 mg L^−1^
q_e_ experimental (mg g^−1^)	112.75	175.98	201.46	212.56
*Pseudo first order*				
q_e_, mg g^−1^	107.65	167.45	183.10	206.99
K_1_ (min^−1^)	0.08087	0.11886	0.1261	0.17078
R^2^	0.9758	0.9632	0.948	0.985
*Pseudo second order*				
q_e_, mg g^−1^	116.97	178.54	203.58	217.59
K_2_ (×10^−3^ g mg^−1^ min^−1^)	1.01	1.10	1.06	1.48
R^2^	0.998	0.989	0.987	0.996

**Table 2:** Isotherm parameters obtained by fitting MB adsorption data for RF/TiO_2_ to the Langmuir, Freundlich, Sips, and Toth equations.

Langmuir	q_m_ (mg g^−1^)	254.65
	K_L_ (L mg^−1^)	0.0732
	R^2^	0.960
Freundlich	K_F_ mg g^−1^ (L mg^-1^)^1/n^	54.85
	n_F_	3.1993
	R^2^	0.865
Sips	q_s_ (mg g^−1^)	218.71
	K_S_	0.010
	n_s_	1.913
	R^2^	0.994
Toth	q_m_ (mg g^−1^)	558.47
	K_T_	0.0295
	n_T_	1.403
	R^2^	0.991

**Table 3:** Thermodynamic data for MB adsorption onto RF/TiO_2_ at various temperatures.

T (K)	lnk	∆G⁰ (KJ/mol)	∆S⁰ (J/mol)	112
281	1.29	−3.01	**∆H⁰ (KJ/mol)**	28.2
296	2.20	−5.41		
305	2.40	−6.09		
313	2.50	−6.51		

## Supplementary Materials

The following supporting information can be downloaded at: https://www.mdpi.com/article/10.3390/gels8040215/s1, Figure S1: Zone of Energy dispersive x-ray (EDX) spectra; Figure S2: Point of zero charge (pHpzc) on the surface of RF/TiO_2_; Table S1: Kinetic parameters obtained by fitting kinetic data for MB adsorption to RF/TiO_2_.; Table S2: Isotherm parameters obtained by fitting MB adsorption data for RF/TiO_2_ to the Langmuir, Freundlich, SIPS and Toth equations.

## References

[1] Xue G., Liu H., Chen Q., Hills C., Tyrer M., Innocent F. (2011). Synergy between surface adsorption and photocatalysis during degradation of humic acid on TiO_2_/activated carbon composites. J. Hazard. Mater..

[2] Ajiboye T.O., Oyewo O.A., Onwudiwe D.C. (2021). Adsorption and photocatalytic removal of Rhodamine B from wastewater using carbon-based materials. FlatChem.

[3] Quyen N.D.V., Khieu D.Q., Tuyen T.N., Tin D.X., Diem B.T.H., Dung H.T.T. (2021). Highly effective photocatalyst of TiO_2_ nanoparticles dispersed on carbon nanotubes for methylene blue degradation in aqueous solution. Vietnam. J. Chem..

[4] Sampaio M.J., Silva C.G., Marques R.R., Silva A.M., Faria J.L. (2011). Carbon nanotube—TiO_2_ thin films for photocatalytic applications. Catal. Today.

[5] Murgolo S., Petronella F., Ciannarella R., Comparelli R., Agostiano A., Curri M.L., Mascolo G. (2015). UV and solar-based photocatalytic degradation of organic pollutants by nano-sized TiO_2_ grown on carbon nanotubes. Catal. Today.

[6] Morawski A.W., Kusiak-Nejman E., Wanag A., Narkiewicz U., Edelmannová M., Reli M., Kočí K. (2021). Influence of the calcination of TiO_2_-reduced graphite hybrid for the photocatalytic reduction of carbon dioxide. Catal. Today.

[7] Minella M., Sordello F., Minero C. (2017). Photocatalytic process in TiO_2_/graphene hybrid materials. Evidence of charge separation by electron transfer from reduced graphene oxide to TiO_2_. Catal. Today.

[8] Faraldos M., Bahamonde A. (2017). Environmental applications of titania-graphene photocatalysts. Catal. Today.

[9] Zeng G., You H., Du M., Zhang Y., Ding Y., Xu C., Liu B., Chen B., Pan X. (2021). Enhancement of photocatalytic activity of TiO_2_ by immobilization on activated carbon for degradation of aquatic naphthalene under sunlight irradiation. Chem. Eng. J..

[10] Paušová M., Riva M., Baudys M., Krýsa J., Barbieriková Z., Brezová V. (2019). Composite materials based on active carbon/TiO_2_ for photocatalytic water purification. Catal. Today.

[11] Khalid N., Majid A., Tahir M.B., Niaz N., Khalid S. (2017). Carbonaceous-TiO_2_ nanomaterials for photocatalytic degradation of pollutants: A review. Ceram. Int..

[12] Biener J., Stadermann M., Suss M., Worsley M.A., Biener M.M., Rose K.A., Baumann T.F. (2011). Advanced carbon aerogels for energy applications. Energy Environ. Sci..

[13] Faria J.L., Wang W. (2009). 13 Carbon Materials in Photocatalysis.

[14] Al-Muhtaseb S., Ritter J. (2003). Preparation and Properties of Resorcinol-Formaldehyde Organic and Carbon Gels. Adv. Mater..

[15] Prostredný M., Abduljalil M.G., Mulheran P.A., Fletcher A.J. (2018). Process variable optimization in the manufacture of resorcinol–formaldehyde gel materials. Gels.

[16] Awadallah-F A., Al-Muhtaseb S.A. (2012). Nanofeatures of resorcinol-formaldehyde carbon microspheres. Mater. Lett..

[17] Awadallah-F A., Elkhatat A.M., Al-Muhtaseb S.A. (2011). Impact of synthesis conditions on meso- and macropore structures of resorcinol-formaldehyde xerogels. J. Mater. Sci..

[18] Principe I.A., Fletcher A.J. (2018). Parametric study of factors affecting melamine-resorcinol-formaldehyde xerogels properties. Mater. Today Chem..

[19] Shevlin S.A., Woodley S.M. (2010). Electronic and Optical Properties of Doped and Undoped (TiO_2_)n Nanoparticles. J. Phys. Chem. C.

[20] Jiang Y., Meng L., Mu X., Li X., Wang H., Chen X., Wang X., Wang W., Wu F., Wang X. (2012). Effective TiO_2_ hybrid heterostructure fabricated on nano mesoporous phenolic resol for visible-light photocatalysis. J. Mater. Chem..

[21] Zaleska A. (2008). Doped-TiO_2_: A review. Recent Pat. Eng..

[22] Aranovich G.L., Donohue M.D. (1995). Adsorption isotherms for microporous adsorbents. Carbon.

[23] Wang S., Zhu Z.H., Coomes A., Haghseresht F., Lu G.Q. (2005). The physical and surface chemical characteristics of activated carbons and the adsorption of methylene blue from wastewater. J. Colloid Interface Sci..

[24] Chham A., Khouya E., Oumam M., Abourriche A., Gmouh S., Mansouri S., Elhammoudi N., Hanafi N., Hannache H. (2018). The use of insoluble mater of Moroccan oil shale for removal of dyes from aqueous solution. Chem. Int..

[25] Atout H., Bouguettoucha A., Chebli D., Gatica J.M., Vidal H., Yeste M.P., Amrane A. (2017). Integration of Adsorption and Photocatalytic Degradation of Methylene Blue Using TiO_2_ Supported on Granular Activated Carbon. Arab. J. Sci. Eng..

[26] Matos J. (2013). Hybrid TiO_2_-C Composites for the Photodegradation of Methylene Blue Under Visible Light.

[27] Natarajan T.S., Bajaj H.C., Tayade R.J. (2014). Preferential adsorption behavior of methylene blue dye onto surface hydroxyl group enriched TiO_2_ nanotube and its photocatalytic regeneration. J. Colloid Interface Sci..

[28] Baker F.S., Miller C.E., Repik A.J., Tolles E.D. (2000). Activated carbon. Kirk-Othmer Encyclopedia of Chemical Technology.

[29] Zhang X., Zhang F., Chan K.-Y. (2005). Synthesis of titania-silica mixed oxide mesoporous materials, characterization and photocatalytic properties. Appl. Catal. A Gen..

[30] Ashraf M.A., Peng W., Zare Y., Rhee K.Y. (2018). Effects of Size and Aggregation/Agglomeration of Nanoparticles on the Interfacial/Interphase Properties and Tensile Strength of Polymer Nanocomposites. Nanoscale Res. Lett..

[31] Azizian S. (2004). Kinetic models of sorption: A theoretical analysis. J. Colloid Interface Sci..

[32] Foo K.Y., Hameed B.H. (2010). Insights into the modeling of adsorption isotherm systems. Chem. Eng. J..

[33] Ayawei N., Ebelegi A.N., Wankasi D. (2017). Modelling and Interpretation of Adsorption Isotherms. J. Chem..

[34] Tan Y., Kilduff J.E. (2007). Factors affecting selectivity during dissolved organic matter removal by anion-exchange resins. Water Res..

[35] Li J., Zhang S., Chen C., Zhao G., Yang X., Li J., Wang X. (2012). Removal of Cu(II) and Fulvic Acid by Graphene Oxide Nanosheets Decorated with Fe_3_O_4_ Nanoparticles. ACS Appl. Mater. Interfaces.

[36] Shao D.D., Fan Q.H., Li J.X., Niu Z.W., Wu W.S., Chen Y.X., Wang X.K. (2009). Removal of Eu(III) from aqueous solution using ZSM-5 zeolite. Microporous Mesoporous Mater..

[37] Zhang G., Ni C., Liu L., Zhao G., Fina F., Irvine J.T.S. (2015). Macro-mesoporous resorcinol–formaldehyde polymer resins as amorphous metal-free visible light photocatalysts. J. Mater. Chem. A.

[38] Chen X., Mao S.S. (2007). Titanium dioxide nanomaterials: Synthesis, properties, modifications, and applications. Chem. Rev..

[39] Huang X., Yang W., Zhang G., Yan L., Zhang Y., Jiang A., Xu H., Zhou M., Liu Z., Tang H. (2019). Alternative synthesis of nitrogen and carbon co-doped TiO_2_ for removing fluoroquinolone antibiotics in water under visible light. Catal. Today.

[40] Simonetti E.A.N., de Simone Cividanes L., Campos T.M.B., de Menezes B.R.C., Brito F.S., Thim G.P. (2016). Carbon and TiO_2_ synergistic effect on methylene blue adsorption. Mater. Chem. Phys..

[41] Wu C.H., Kuo C.Y., Chen S.T. (2013). Synergistic effects between TiO_2_ and carbon nanotubes (CNTs) in a TiO_2_/CNTs system under visible light irradiation. Environ. Technol..

[42] Houas A., Lachheb H., Ksibi M., Elaloui E., Guillard C., Herrmann J.M. (2001). Photocatalytic degradation pathway of methylene blue in water. Appl. Catal. B: Environ..

[43] Lakshmi S., Renganathan R., Fujita S. (1995). Study on TiO_2_-mediated photocatalytic degradation of methylene blue. J. Photochem. Photobiol. A Chem..

[44] Makuła P., Pacia M., Macyk W. (2018). How To Correctly Determine the Band Gap Energy of Modified Semiconductor Photocatalysts Based on UV–Vis Spectra. J. Phys. Chem. Lett..

[45] Ho Y.S., McKay G. (1999). Pseudo-second order model for sorption processes. Process Biochem..

[46] Sahoo T.R., Prelot B. (2020). Adsorption processes for the removal of contaminants from wastewater: The perspective role of nanomaterials and nanotechnology. Nanomaterials for the Detection and Removal of Wastewater Pollutants.

