# Supplementary material for: Effective Carbon/TiO2 Gel for Enhanced Adsorption and Demonstrable Visible Light Driven Photocatalytic Performance
Source: Gels. 2022 Apr 1;8(4):215. doi: 10.3390/gels8040215 (PMC9031654; doi:10.3390/gels8040215)
Supplement: Supplementary file 1 [file gels-08-00215-s001.zip › gels-1614498-supplementary.pdf]

Effective carbon/TiO_2 gel for enhanced adsorption and demonstrable visible light driven photocatalytic performance

Anam Safri and Ashleigh Fletcher*

Supporting Information

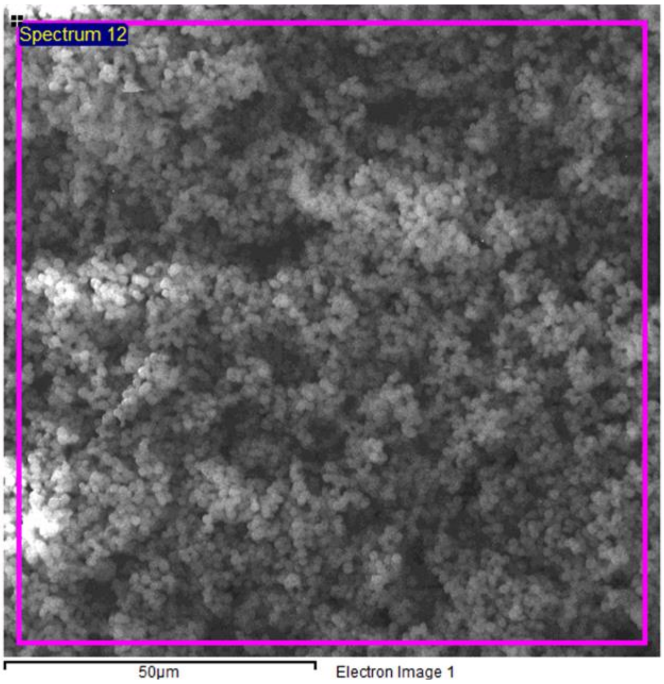

Figure S1: Zone of Energy dispersive x-ray (EDX) spectra

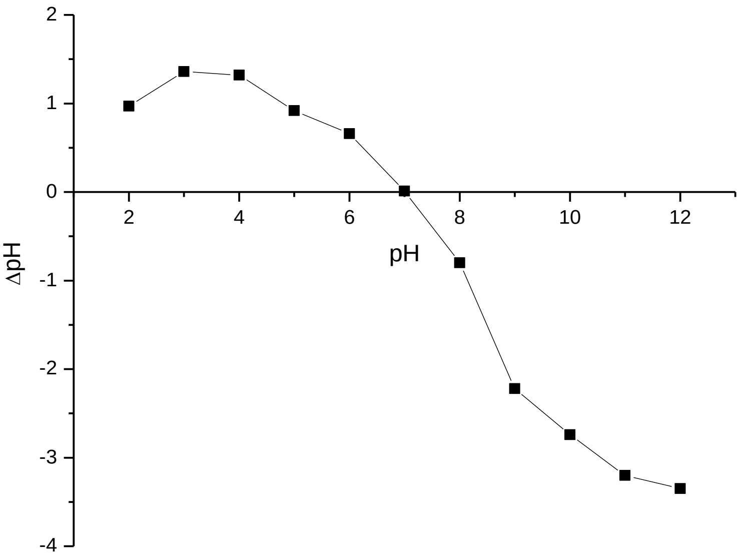

Figure S2: Point of zero charge (pHpzc) on the surface of RF/TiO_2

Table S1. Kinetic parameters obtained by fitting kinetic data for MB adsorption to RF/TiO_2.

Model	50 mg L^{-1}	100 mg L^{-1}	150 mg L^{-1}	200 mg L^{-1}
**q_e experimental (mg g^{-1})**	112.7	175.9	201.5	212.6
Pseudo first order				
**q_e, mg g^{-1}**	107.7	167.5	183.1	206.9
**K_1 (min $^{-1}$)**	0.081	0.119	0.126	0.171
**R^2**	0.975	0.963	0.948	0.985
**ARE**	-0.524	0.888	9.387	-2.535
**SSE**	490.3	674.1	3452	572.9
**RMSE**	7.002	8.210	18.58	7.569
**HYBRID**	0.036	-0.061	-0.647	0.174
**MPSD**	3.339	5.654	59.74	16.13
**Chi square**	5.325	6.504	21.69	3.713
Pseudo second order				
**q_e, mg g^{-1}**	116.9	178.54	203.6	217.59
**K^2 (x 10^{-3} g mg^{-1} min^{-1})**	1.01	1.10	1.06	1.48
**R^2**	0.998	0.989	0.987	0.996
**ARE**	-2.770	0.113	4.432	-2.949
**SSE**	817.6	505.3	1492	856.5
**RMSE**	9.042	7.108	12.21	9.255
**HYBRID**	0.191	-0.007	-0.305	0.203
**MPSD**	17.63	0.719	28.21	18.77
**Chi square**	9.068	3.726	8.589	5.124

Table S2. Isotherm parameters obtained by fitting MB adsorption data for RF/TiO_2 to the Langmuir, Freundlich, SIPS and Toth equations

**Langmuir**	q_m (mg g^{-1})	254.7
	K_L (L mg^{-1})	0.073
	ARE	-16.69
	SSE	3566
	RMSE	36.64
	HYBRID	19.91
	MPSD	-20.87
	Chi square	118.0
	R^2	0.960
**Freundlich**	K_F mg g^{-1} $(L/mg)^{1/n}$	54.85
	n_F	3.199
	ARE	-21.25
	SSE	6149
	RMSE	27.72
	HYBRID	-26.56
	MPSD	150.3
	Chi square	86.43
	R^2	0.865
**SIPS**	q_s (mg g^{-1})	218.7
	K_S	0.010
	n_s	1.913
	ARE	-9.675
	SSE	3930
	RMSE	22.17
	HYBRID	-12.09
	MPSD	68.41
	Chi square	31.21
	R^2	0.994
**Toth**	q_m (mg g^{-1})	558.5
	K_T	0.029
	n_T	1.403
	ARE	-13.83
	SSE	4033
	RMSE	22.45
	HYBRID	-17.28
	MPSD	97.76
	Chi square	29.99
	R^2	0.991
